# Exchange bias in ferromagnetic bilayers with orthogonal anisotropies: the case of GaMnAsP/GaMnAs combination

**DOI:** 10.1038/s41598-019-49492-4

**Published:** 2019-09-10

**Authors:** Suho Choi, Seul-Ki Bac, Xinyu Liu, Sanghoon Lee, Sining Dong, M. Dobrowolska, J. K. Furdyna

**Affiliations:** 1 Department of Physics, Korea University, Seoul, 136-701 Korea; 2 Department of Physics, University of Notre Dame, Notre Dame, Indiana, 46556 USA

**Keywords:** Ferromagnetism, Magnetic properties and materials, Semiconductors, Spintronics, Surfaces, interfaces and thin films

## Abstract

We report the observation of exchange bias in a ferromagnetic Ga_0.94_Mn_0.06_As_0.77_P_0.23_/ Ga_0.94_Mn_0.06_As bilayer, in which the easy axis in one layer is oriented out-of-plane, and in the other in-plane. Magnetization reversal in this system is explored using planar Hall effect (PHE) measurements under various initial conditions and with various field-cooling orientations. Our results show that the two magnetic layers are ferromagnetically exchange-coupled, and that such coupling results in pronounced exchange-bias-like shifts of magnetic hysteresis loops during reversal of in-plane magnetization. The presence of exchange bias in this system can be understood on the basis of magnetic closure domains formed in the layer with the out-of-plane easy axis.

## Introduction

Ferromagnetic (FM) films with easy axes normal to the film plane have been extensively studied because of their promise for applications in perpendicular magnetic recording media^1,2^. It is well established that magnetic anisotropy in the FM semiconductor (Ga_,_Mn)As can be manipulated by alloying this material with phosphorus (P), which modifies strain conditions of the film in a way that results in an easy axis normal to the film when the concentration of P is sufficiently high^3–5^. By fabricating Ga_0.94_Mn_0.06_As_0.77_P_0.23_/Ga_0.94_Mn_0.06_As bilayers on GaAs (100) substrates, one can therefore, obtain monolithic FM structures consisting of two dissimilar magnetic layers, with a common in-plane lattice parameter, but with magnetizations at right angles to one another, thus providing a model system for investigating the behavior of orthogonal interfacial exchange coupling in FM semiconductor systems^2,6^.

**Figure 1:**
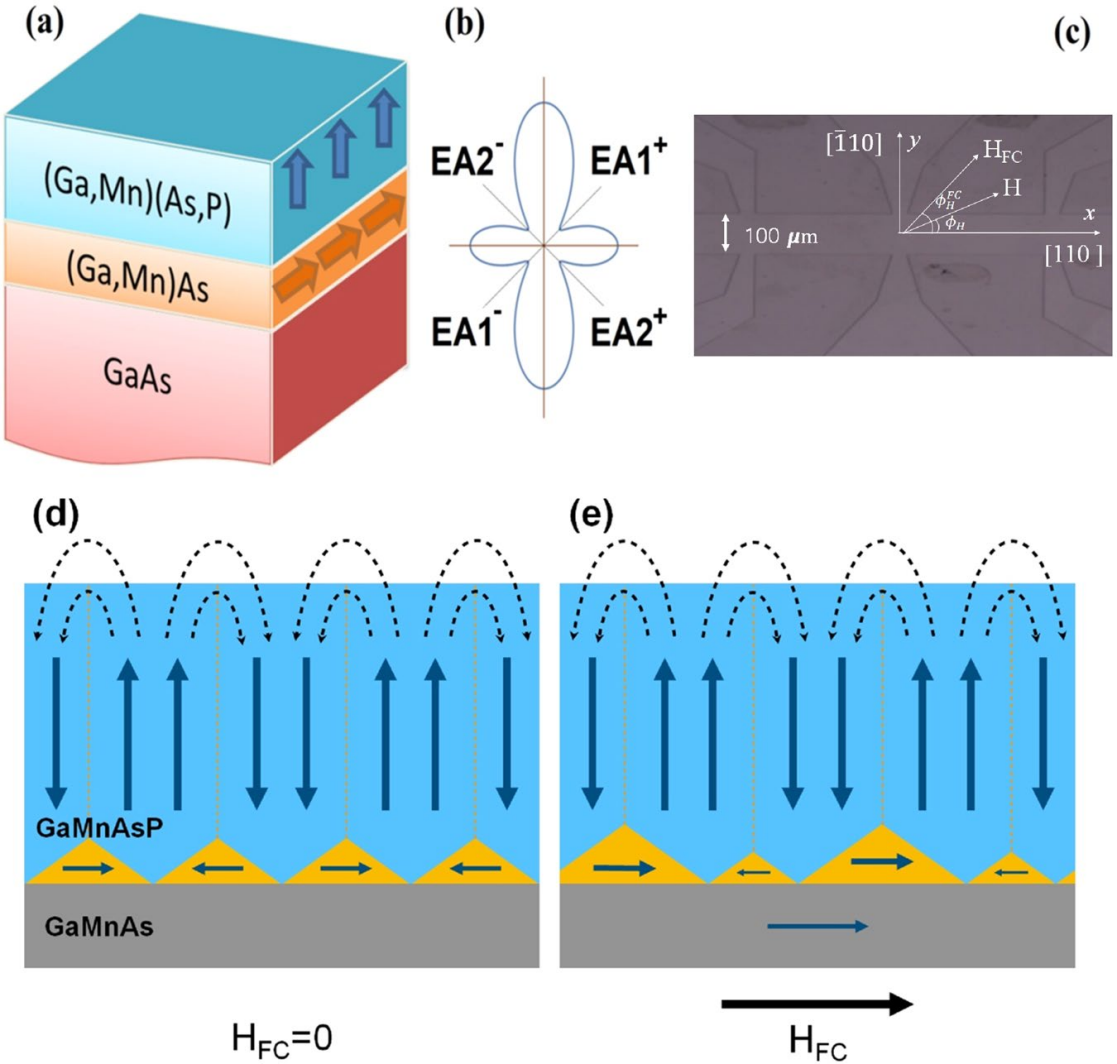
(**a**) Schematic diagram of the Ga_0.94_Mn_0.06_As_0.77_P_0.23_(21 nm)/Ga_0.94_Mn_0.06_As(12.5 nm) bilayer grown on GaAs (100) substrate. (**b**) Polar plot of in-plane magnetic free energy of the Ga_0.94_Mn_0.06_As layer, showing the four easy magnetization directions. (**c**) Optical image of Hall bar for planar Hall resistance measurement. (**d**) Closure domain at the Ga_0.94_Mn_0.06_As_0.77_P_0.23_/Ga_0.94_Mn_0.06_As interface, accommodating the closure of domains in GaMnAsP magnetized normal to the film plane in zero field. (**e**) Closure domains after field cooling, resulting in exchange bias, as discussed in the text.

One interesting phenomenon in such orthogonally-coupled FM bilayers is the occurrence of exchange bias effect in these geometries^7–12^. This effect is different from the more familiar exchange bias resulting from interfacial exchange interaction between a ferromagnetic and an antiferromagnetic material^13^, and can be explained as a consequence of the interplay between the out-of-plane and the in-plane anisotropies of the two adjacent FM films^14^. It is suggested that closure domains in the layer with the easy axis normal to the film are created in the region close to the interface between the two coupled FM layers, as shown schematically in Fig. 1(d–e). In micromagnetic simulations^15^, such closure domains with magnetizations oriented parallel and anti-parallel to an applied magnetic field change their respective volumes, and the resulting net in-plane magnetic moment of the closure domains then induces an exchange bias in the FM layer magnetized in-plane.

Encouraged by these expectations, we grew a series of Ga_0.94_Mn_0.06_As_0.77_P_0.23_/Ga_0.94_Mn_0.06_As bilayers on GaAs (100) substrates by low-temperature molecular beam epitaxy^16^. The structure of the bilayer is shown schematically in Fig. 1(a). In order to further understand the interface exchange coupling mechanisms between the two FM layers, we carried out a comprehensive study of magnetization reversal in these Ga_0.94_Mn_0.06_As_0.77_P_0.23_/ Ga_0.94_Mn_0.06_As bilayers using planar Hall effect (PHE) measurements. Our results show that the Ga_0.94_Mn_0.06_As_0.77_P_0.23_ and Ga_0.94_Mn_0.06_As layers are indeed orthogonally exchange-coupled, as evidenced by the observation of pronounced shifts of the PHE loops of the Ga_0.94_Mn_0.06_As layer described in what follows.

## Experiment

The growth of Ga_0.94_Mn_0.06_As_0.77_P_0.23_/Ga_0.94_Mn_0.06_As bilayers was carried out as follows. The Ga_1-*x*_Mn_*x*_As layer was grown first to a thickness of 12 nm with the phosphorus cracker cell closed. The phosphorus cell was then opened, and a ~21 nm Ga_0.94_Mn_0.06_As_0.77_P_0.23_ layer was deposited directly on Ga_0.94_Mn_0.06_As. The thicknesses of 12 nm and 21 nm for Ga_0.94_Mn_0.06_As and Ga_0.94_Mn_0.06_As_0.77_P_0.23_ layers, respectively, were selected in order to have well-defined in-plane easy axes in Ga_1-*x*_Mn_*x*_As and out-of-plane easy axes in Ga_0.94_Mn_0.06_As_0.77_P_0.23_^17^, so that we can investigate the interaction between orthogonal magnetic configuration in Ga_0.94_Mn_0.06_As_0.77_P_0.23_/Ga_0.94_Mn_0.06_As_0.77_ bilayers. During the growth the Mn content was kept constant in both layers at ~0.06. The P content in the Ga_0.94_Mn_0.06_As_0.77_P_0.23_ layer was selected in order to achieve a large tensile strain, to make certain that the easy axis in that layer was normal to the layer plane^5,16^. The growth was monitored by reflection high energy electron diffraction (RHEED), which showed a streaky (1 × 2) RHEED pattern throughout the growth of both layers, indicating high crystalline quality of the samples.

Temperature dependences of in-plane and out-of-plane magnetizations on the Ga_0.94_Mn_0.06_As_0.77_P_0.23_/Ga_0.94_Mn_0.06_As bilayer were carried out using a magnetometer; the Curie temperatures *T*_*c*_ of the Ga_0.94_Mn_0.06_As and the Ga_0.94_Mn_0.06_As_0.77_P_0.23_ were identified as 70 K and 45 K, respectively. Measurement of the planar Hall resistance (PHR) in magnetic fields whose direction can be applied along arbitrary in-plane direction provides a particularly convenient tool for investigating the details of magnetization reversal in the layer plane^18,19^, and we have adapted this approach for investigating the effects of exchange bias in the Ga_0.94_Mn_0.06_As layer^20^. For this purpose the sample was patterned by photolithography and by chemical wet etching into 100 μm × 1500 μm Hall bars with the long dimension lying along the [110] direction, as shown in Fig. 1(c). Measurements of the planar Hall resistance *R*_*xy*_ were performed by using a sample holder and experimental arrangement such that a magnetic field could be applied in the plane of the sample at an arbitrary angle. The experiments consisted of measuring the dependence of the planar Hall resistance (PHR) on the external field swept in several selected field directions, after cooling fields of various strengths and orientations were applied to the bilayer. The angles of magnetization *ϕ*_*M*_, of the applied magnetic field *ϕ*_*H*_, and of the cooling field *ϕ*_*H*_^*FC*^ are measured counterclockwise from the [110] crystallographic direction in the (001) plane.

## Results

The in-plane anisotropy of the Ga_0.94_Mn_0.06_As layer were determined by measuring the angular dependence of PHR and by analyzing the results based on conditions that minimize magnetic free energy during the rotation of magnetization, as described by Son *et al*.^21^. This method is now well established as a tool for investigating magnetic anisotropy of ferromagnetic films. The obtained magnetic anisotropy energy density for the Ga_0.94_Mn_0.06_As layer is plotted in Fig. 1(b), showing the in-plane easy axes near the <100> directions.

### Cooling field dependence of PHE

**Figure 2:**
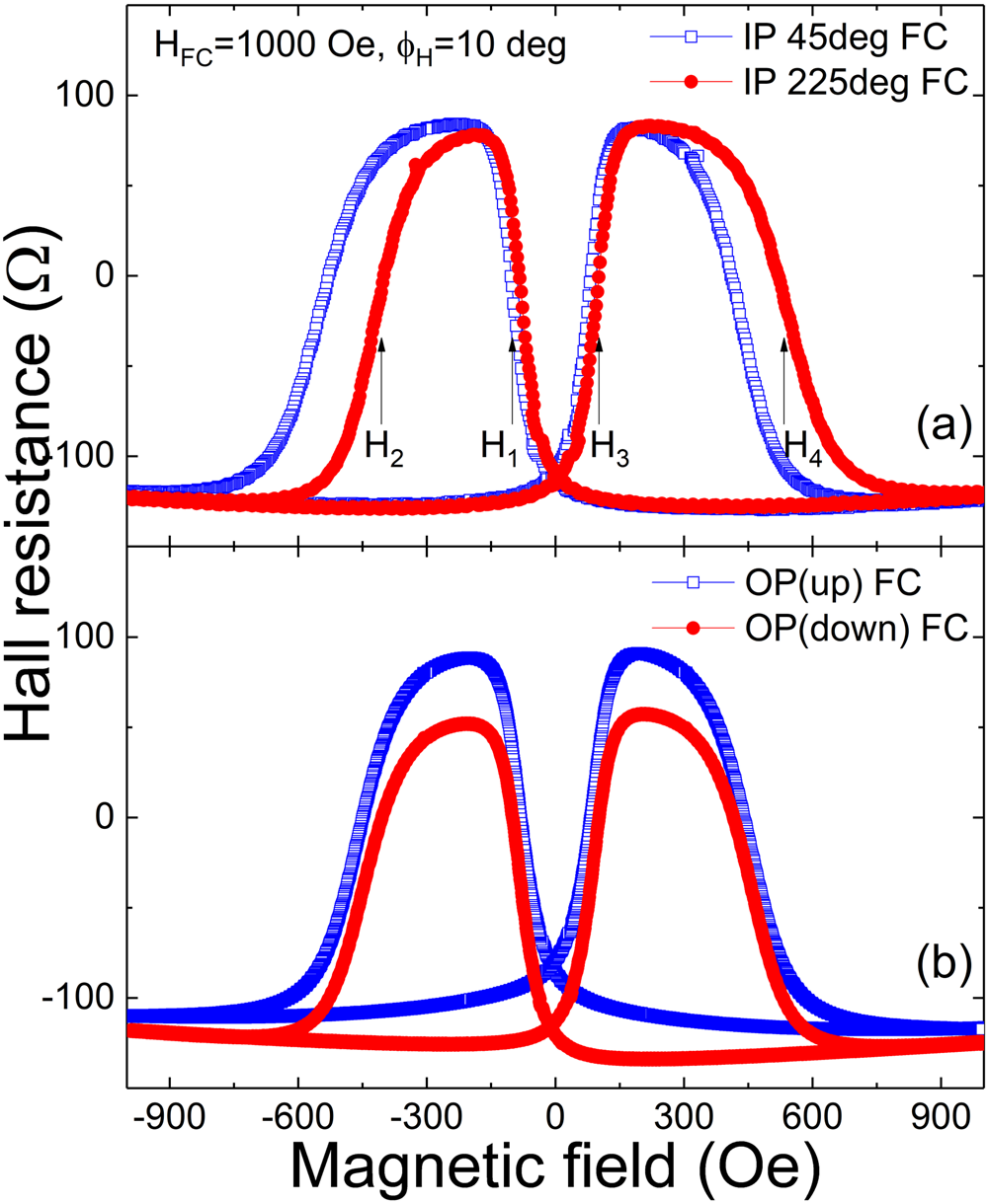
(**a**) Two hysteresis loops of PHR data obtained after the sample was cooled to 3 K in a field of 1 kOe applied either at 45° or 225° with respect to the [110] direction. (**b**) Two hysteresis loops of PHR data obtained after the sample was cooled to 3 K in a field of 1 kOe applied perpendicular, either up or down, with respect to the sample plane.

Figure 2(a) shows results of PHR measurements at 3 K during magnetization reversal scans for several field orientations. To discuss these results, it is important to describe the sequence of steps used in taking the data. The sample was cooled to 3 K in a field of 1 kOe applied in-plane either at *ϕ*_*H*_^*FC*^ = 45° or at 225° with respect to the [110] direction. After reducing the field to zero, the magnet was rotated to *ϕ*_*H*_ = 10°, as indicated in the figure, and the PHR was measured during a field scan between −1000 Oe and 1000 Oe. Qualitatively, the behavior of PHR scan shown in Fig. 2 is typical for Ga_0.94_Mn_0.06_As, showing abrupt changes of sign when magnetization experiences quasi-90° reorientations between the two in-plane easy axes in adjacent quadrants as the field is swept. This behavior can be described by the equation^22^

1 $${{\rm{R}}}_{{\rm{PHR}}}=(k/t){{\rm{M}}}^{2}\,\sin (2{\varphi }_{M}),$$

where *t* is sample thickness, *k* is a negative constant related to anisotropic magnetoresistance, and *ϕ*_*M*_ is the angle between the current (*i*.*e*., the [110] direction) and magnetization.

The most interesting feature of the hysteresis shown in Fig. 2(a) is the conspicuous asymmetry of the PHR data obtained after in-plane field cooling, indicating exchange-bias-like behavior. Marking the fields at which magnetization switches from one easy axis to the next during magnetization reversal as *H*_1_ … *H*_4_, as shown in Fig. 2, the PHR asymmetry is conveniently expressed by the differences of the switching fields |*H*_1_|-|*H*_3_| and |*H*_2_|-|*H*_4_|.

In Fig. 2(b), the sample was cooled to 3 K in a field of 1 kOe applied normal to the sample plane, either up or down. The field was then brought to zero, and PHR was measured during in-plane field scans between −1000 Oe and 1000 Oe at *ϕ*_*H*_ = 10°, as in Fig. 2(a). In this case the hysteresis loops of PHR are symmetric, but with a vertical shift. The data in Fig. 2 thus reveal that exchange-bias-like shifts are only observed after cooling in an in-plane field.

### PHE and exchange bias

**Figure 3:**
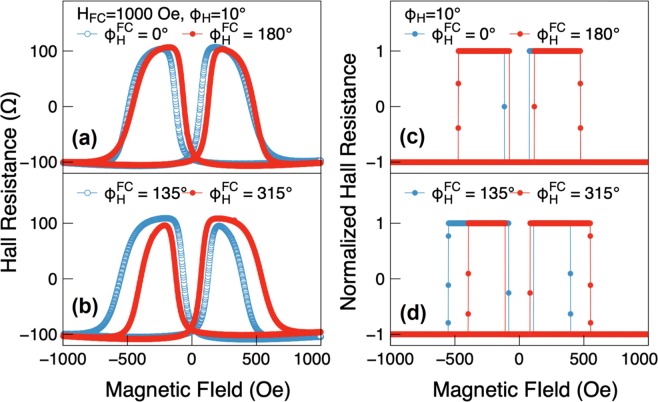
(Left column) Hysteresis loops of PHR data obtained after the sample was cooled to 3 K in an in-plane field of 1 kOe applied in various angles with respect to the [110] direction. (Right column) Results of simulation for the PHR during magnetization reversal obtained with *H*_*EB*_ = 38 Oe. The asymmetric behavior of magnetization transitions shown in the left column is nicely reproduced in the simulation on the right.

In Fig. 3(a,b) we plot the hysteresis loops of PHR for field sweeps along *ϕ*_*H*_ = 10° after applying the cooling field in several in-plane directions. From the data we conclude that the exchange bias of our bilayer film depends on the orientation of the cooling field. The observed behavior of PHR can be readily understood by considering the free energy of the (Ga,Mn)As layer that includes the additional bias field *H*_*EB*_ oriented at some angle *ϕ*_*EB*_ in the layer plane. This can be written as^19^

2 $$\frac{E}{M}=\frac{{H}_{c}}{8}{\cos }^{2}2{\varphi }_{M}+\frac{{H}_{u}}{2}{\sin }^{2}{\varphi }_{M}-{H}_{EB}\,\cos ({\varphi }_{M}-{\varphi }_{EB})-H\,\cos ({\varphi }_{M}-{\varphi }_{H}),$$

where *H* is the external magnetic field; *H*_*c*_ and *H*_*u*_ are the cubic and uniaxial anisotropy fields; and *H*_*EB*_ is the exchange bias field. The angles *ϕ*_*M*_, *ϕ*_*H*_, and *ϕ*_*EB*_ are the directions of magnetization *M*, the applied magnetic field *H*, and the exchange bias field *H*_*EB*_, respectively, measured counterclockwise from the [110] direction. In our experiment we assume that the cooling field imprints the field *H*_*EB*_ along its direction, so that *ϕ*_*EB*_ = *ϕ*_*H*_^*FC*^.

**Figure 4:**
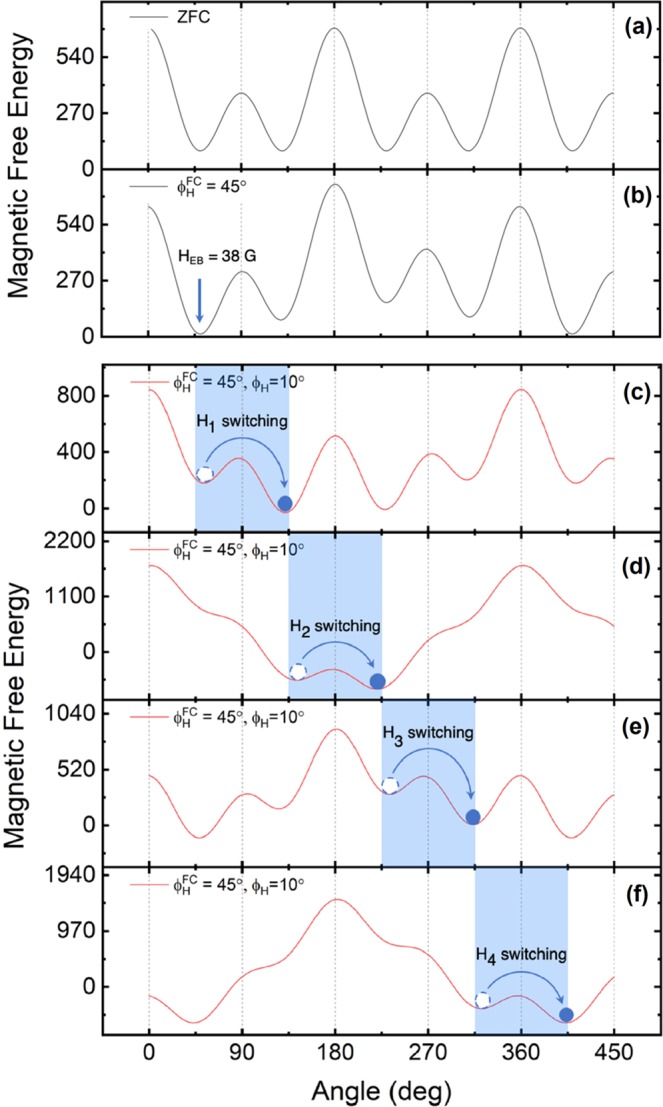
Magnetic free-energy density of the film without (**a**) and with *H*_*EB*_ = 38 Oe (**b**) The presence of an exchange bias field distorts the energy profile, making it asymmetric with respect to the crystallographic directions. (**c**–**f)** Magnetic free-energy density profile at the moment of rotation of magnetization corresponding to the switching fields *H*_1_, *H*_2_, *H*_3_ and *H*_4_. The relative change of the energy minima and maxima can be clearly seen at each switching field. The energy minima involving each transition are marked with shaded region. Open dots at the minima indicate the orientation of the initial state of magnetization, and solid dots indicate its final state after reorientation occurs.

The free energy, Eq. (2), determines the four easy magnetization directions of the Ga_0.94_Mn_0.06_As film in the layer plane with respect to the [110] crystal axis, shown at 45° + δ, 135°−δ, 225° + δ, and 315°−δ in Fig. 4(a). Here the *δ* is a deviation angle of magnetic easy axes from the <100> directions. We have plotted the energy profiles calculated at zero applied field *H* = 0, using the cubic and uniaxial anisotropy parameters in the absence of exchange bias. Since the energy profile at *H* = 0 is symmetric with respect to the <110> crystallographic directions, magnetization transitions between the easy axes are also symmetric with respect to the <110> directions. This is seen in Fig. 2(b), obtained when the sample was cooled in a field applied normal to the sample plane, *i*.*e*., when there is no exchange bias, as discussed later in the paper. It is readily seen from Eq. (2) that when the field is swept along some direction, the values of the free energy minima change relative to one another. In particular, when the magnetization lies initially along the energy minimum in quadrant, and the energy minimum in the adjacent quadrant is made deeper by the applied field, the magnetization will switch to the lower minimum when the difference between the initial and the final minimum exceeds a domain pinning energy^23,24^. By inserting the switching fields observed for this symmetric results shown in Fig. 2(b), we can then determine the difference between two energy minima required for the magnetization transition to occur in the absence of *H*_*EB*_.

The energy profile is no longer symmetric with respect to the < 110 > directions when *H*_*EB*_ is present in the sample plane, as seen in Fig. 4(b) (*H*_*EB*_ = 38 Oe and *ϕ*_*EB*_ = 45°). However, for magnetization transitions to occur between adjacent quadrants, the same conditions on the difference between adjacent energy minima are expected to apply as in the symmetric case. By varying the strength of *H*_*EB*_, we can then simulate the behavior of such asymmetric transitions obtained for different field cooling directions. The best results of simulation for several field cooling directions are obtained with *H*_*EB*_ = 38 Oe, and are plotted in Fig. 3(c,d). The simulation nicely describes the asymmetric behavior of switching fields that is observed in the experiment (see the corresponding panels in the left column of Fig. 3).

Additionally, when a magnetic field is applied along 10°, that further distorts the free energy profile by making the energy of some minima deeper and maxima weaker, and others higher. This is illustrated in Fig. 4(c–f), where we show the energy profile at the four fields corresponding to transitions of magnetization calculated *H*_1_, *H*_2_, *H*_3_ and *H*_4_ as the applied field is swept along *ϕ*_*H*_ = 10°.

The specific case illustrated in Fig. 4(c–f) is as follows: the field is initially swept along *ϕ*_*H*_ = 10°, which has a component along the easy axis in the Quadrant 1, and orients the magnetization along that axis (near 45°). The field is then reversed. As the field increases in the reverse direction, its projection on the easy axis in Quadrant 2 first “flips” the magnetization to that easy axis as the minimum in Quadrant 2 is deep enough to overcome the intervening barrier, and finally flips it again to the easy axis in Quadrant 3. As the field is then swept in the positive direction, in a similar sequence the magnetization first “flips” to Quadrant 4, and finally back to Quadrant 1. Note, however, that the magnetization transition at *H*_4_ to the easy axis in Quadrant 1 occurs at a lower field than the transition at *H*_2_, in Quadrant 3 because the projection of the applied field causing the transitions is aided by the bias field in Quadrant 1, while it is opposed by the presence of the bias field in Quadrant 3. This is the cause of the observed asymmetry in PHR. The cases with *H*_*EB*_ oriented in other directions, shown in Fig. 3(a,b) can be similarly analyzed.

### Cooling field and temperature dependences of exchange bias

**Figure 5:**
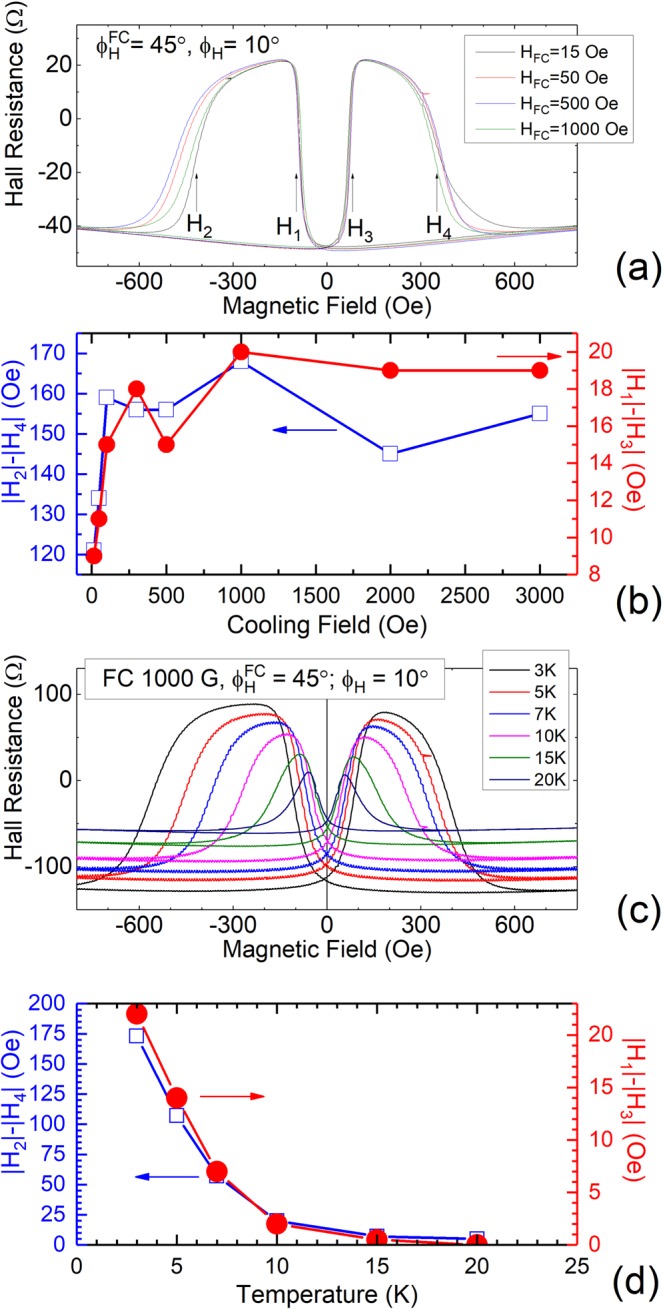
(**a**) The hysteresis loops of PHR data obtained under different magnitudes of cooling field. (**b**) The shifts observed in (**a**) plotted as function of the magnitude of cooling field. (**c**) Hysteresis loops of PHR data plotted for various temperatures. (**d**) The shifts shown in (**c**) plotted as function of the temperature.

For completeness, we carried out PHR measurements at 3 K using cooling fields of different magnitude. Figure 5(a) shows representative PHR hysteresis loops obtained for four different cooling fields. The asymmetry of the PHR hysteresis curves systematically increases as the cooling field strength is increased. In order to quantify this asymmetry, the differences of transition fields |*H*_1_|-|*H*_3_|and|*H*_2_|-|*H*_4_|are plotted in Fig. 5(b). It is clear from the figure that as the cooling field increases, the exchange bias first increases rapidly, but eventually levels off at fields over ~100 Oe. These results indicate that a moderate cooling field of a few hundred Oe is sufficient to generate an exchange bias in our structure.

Additionally, we have also studied the temperature dependence of exchange bias by using a cooling field of the same value (1.0 kOe), and cooling the system to a series of different temperatures. The data of this series of measurements are plotted in Fig. 5(c). Clearly, the asymmetry of PHR gradually decreases with increasing temperature, with the hysteresis becoming symmetric at temperatures over 20 K. In order to see the temperature dependence of the asymmetry, the differences of transition fields|*H*_1_|-|*H*_3_|and |*H*_2_|-|*H*_4_|obtained at several temperatures are plotted in Fig. 5(d). The asymmetries of the observed PHR scans decrease monotonically at similar rates for both |*H*_1_|-|*H*_3_| and |*H*_2_|-|*H*_4_|as the temperature increases, indicating that the exchange bias in the Ga_0.94_Mn_0.06_As_0.77_P_0.23_/Ga_0.94_Mn_0.06_As bilayer is strong function of temperature.

## Discussion

The observed exchange bias can be explained in terms of closure domains created in the region at the top of the Ga_0.94_Mn_0.06_As_0.77_P_0.23_ layer, as suggested schematically in Fig. 1(d,e). Due to its out-of-plane anisotropy, under in-plane field-cooling conditions, the magnetic structure in Ga_0.94_Mn_0.06_As_0.77_P_0.23_ layer is expected to occur in multi-domain form comprised of out-of-plane and in-plane configurations that satisfy magnetic flux closure requirements indicated in the figure, similar to that observed in metallic systems involving out-of-plane films^12^. The presence of in-plane components of magnetization in the closure domains occurring at Ga_0.94_Mn_0.06_As_0.77_P_0.23_ interfaces then provides a mechanism for exchange coupling with the in-plane magnetization of the Ga_0.94_Mn_0.06_As layer^14^. Specifically, when the Ga_0.94_Mn_0.06_As_0.77_P_0.23_/Ga_0.94_Mn_0.06_As bilayer is cooled in an in-plane field, the closure domains at the interfaces of the Ga_0.94_Mn_0.06_As_0.77_P_0.23_ layer oriented parallel and anti-parallel to the cooling field change their respective sizes, as shown Fig. 1(e), so that the net in-plane magnetic moment of the closure domains induces an exchange bias effect on the hysteresis loop in in-plane phase.

Finally, we note that while the exchange bias effect occurs when the cooling field is applied in-plane, no exchange bias is observed when the cooling field is applied out-of-plane, as seen in Fig. 2(b). This can be understood as follows. When the structure is cooled in an out-of-plane field, the entire Ga_0.94_Mn_0.06_As_0.77_P_0.23_ layer will be magnetized normal to the film, either up or down, depending on the direction of the field. There is then no in-plane magnetic component in the Ga_0.94_Mn_0.06_As_0.77_P_0.23_ layer to couple to the in-plane magnetization of the Ga_0.94_Mn_0.06_As layer. In that situation the Ga_0.94_Mn_0.06_As_0.77_P_0.23_ layer will only contribute to the Hall resistance through the anomalous Hall effect, without coupling. This explains why PHR of Ga_0.94_Mn_0.06_As_0.77_P_0.23_/Ga_0.94_Mn_0.06_As bilayers after out-of-plane field cooling shows only up- and down-shifts of the PHR value, without any asymmetry of the PHR hystereses in the direction of the applied in-plane field, as is indeed seen in Fig. 2(b).

## Supplementary information

Supplementary Information
