# Supplementary material for: Exchange bias in ferromagnetic bilayers with orthogonal anisotropies: the case of GaMnAsP/GaMnAs combination
Source: Sci Rep. 2019 Sep 10;9:13061. doi: 10.1038/s41598-019-49492-4 (PMC6736936; doi:10.1038/s41598-019-49492-4)
Supplement: Supplementary file 1 — Supplementary Information [file 41598_2019_49492_MOESM1_ESM.docx]

**Supplementary Information**

**Exchange bias in ferromagnetic bilayers with orthogonal anisotropies: the case of GaMnAsP/GaMnAs combination**

Suho Choi^1^, Seul-Ki Bac^1,2^, Xinyu Liu^2*^, Sanghoon Lee^1#^, Sining Dong^2^, M. Dobrowolska^2^, and J. K. Furdyna^2^

^1^Department of Physics, Korea University, Seoul, 136-701, Korea

^2^Department of Physics, University of Notre Dame, Notre Dame, Indiana 46556, USA

**Fig. S1** High resolution X-ray diffraction ω-2θ scan with (004) reflection from GaMnAs film studied in this paper.
